# Impact of Short-Time Micronization on Structural and Thermal Properties of Sugar Beet Fibre and Inulin

**DOI:** 10.17113/ftb.60.04.22.7734

**Published:** 2022-12

**Authors:** Miljana Djordjević, Rita Ambrus, Nikola Maravić, Senka Vidović, Dragana Šoronja-Simović, Jovana Petrović, Zita Šereš

**Affiliations:** 1 University of Novi Sad, Faculty of Technology Novi Sad, Department of Carbohydrate Food Engineering, Blvd. cara Lazara 1, 21000 Novi Sad, Serbia; 2 Institute of Pharmaceutical Technology and Regulatory Affairs, University of Szeged, Eötvös street 6, 6720 Szeged, Hungary; 3 University of Novi Sad, Faculty of Technology Novi Sad, Department of Biotechnology and Pharmaceutical Engineering, Blvd. cara Lazara 1, 21000 Novi Sad, Serbia

**Keywords:** superfine grinding, ball milling, dietary fibre, sugar beet pulp, FTIR, XRD

## Abstract

**Research background:**

By tailoring dietary fibre’s structural and physicochemical properties, their functionality and applicability can be remarkably increased. One of the approaches used in this respect is fibre particle size reduction. Accordingly, the present study explores the impact of short-time micronization in a planetary ball mill on structural and thermal changes of modified and commercial sugar beet fibre, inulin and sucrose for their potential application as food excipients.

**Experimental approach:**

Short-time micronization in a planetary ball mill (30 and 60 min) was applied for particle size reduction of modified and commercial sugar beet fibre, inulin and sucrose as less energy-consumptive and less destructive approach than long-time micronization. Dietary fibre and sucrose samples were characterised in terms of particle size, morphology, intermolecular bonds and presence of functional groups, crystallinity and thermal properties, before and after the short-time micronization.

**Results and conclusions:**

Particle size was successfully reduced to micron-scale already after 30 min of micronization in most of the samples without significant changes in thermal properties and crystallinity or present functional groups. An enhanced particle size decrease with prolonged micronization time (60 min) was noticed in modified sugar beet fibre with slightly wider particle size distribution than in other examined samples. Furthermore, morphology and exposure of the present functional groups in samples were altered by the micronization, which is favourable for their further application as excipients in the food matrix.

**Novelty and scientific contribution:**

The corresponding research reports the short-time micronization impact on sugar beet fibre and modified sugar beet fibre, inulin and sucrose for the first time, hence contributing to the widening of their application as excipients in diverse products.

## INTRODUCTION

Dietary fibre represents a vast complex group of polysaccharides, oligosaccharides and associated compounds naturally present in plants. As a step forward to sustainability, food industry by-products (peel, pulp and core) have emerged as dietary fibre sources with far-reaching positive effects contributing to the environment as well as human wellbeing (*1*, *2*). One of the frequently investigated by-products in this respect is the sugar beet pulp remaining after sucrose extraction (*3*-*6*). Sugar beet pulp comprises of soluble (pectin) and insoluble dietary fibre (hemicelluloses, cellulose and lignin), accounting for total dietary fibre content in the range of 74.0‒84.4% (*7*). Additionally, a well-balanced soluble/insoluble dietary fibre ratio, low phytate level and exceptional hydration properties favour the application of sugar beet pulp in the food industry (*8*, *9*). Nevertheless, in order to utilize the full potential of the dietary fibre, they need to be in an assimilable form and as accessible as possible. For that purpose, various mechanical treatments can be employed to decrease the particle size, among which micronization, or superfine grinding, has gained widespread use due to simple handling, maintenance and absence of the detrimental effect on the environment (*10*-*12*). The benefits of micronization are multiple, from tailoring specific dietary fibre physicochemical properties (*10*) and increasing the extent of its physiological function (*13*) to better incorporation within the food matrix and homogenisation with other present ingredients. Additionally, the role of dietary fibre as an excipient in emulsion stabilization and targeted delivery as well as enhancement in dissolution rate primarily relies on particle size distribution. Equipment such as ball mill is frequently used for achieving the desired micronization level (*6*, *11*, *14*, *15*). The main principle of ball mill is the action of pressure, collision and attrition caused by the centrifugal force (*12*, *16*). The control of the micronization intensity is enabled through operational parameter adjustments such as milling speed and time, ball/powder ratio and milling material volume (*12*). Most of the studies employing ball mill for dietary fibre micronization were conducted under milling time 4‒15 h known as long-time milling (long-time micronization), where structural alteration and component redistribution are achieved (*6*, *15*, *17*-*19*). However, fewer studies investigated milling time in the range of 5‒90 min, regarded as short-time milling (short-time micronization) and the corresponding impact on dietary fibre was different depending on the nature of the starting by-product rich in dietary fibre (*14*, *20*). Recently, Lin *et al.* (*21*) subjected sugar beet pulp to short-time micronization by using harsh thermal pre-treatment and ultrasonication, which resulted in softer particle structure and reduction in particle size. Previously, Huang *et al.* (*6*) reported the long-time micronization effect (5 h) on the sugar beet pulp by assessing the particle size distribution, colour difference, physical (bulk and tap density, angle of repose and slide) and hydration properties (water- and oil-binding capacity), thermal characteristics and crystallographic structure. Regarding the short-time micronization by ball mill, effects on sugar beet pulp were not investigated, especially in its chemically modified form. Therefore, the presented study explored the effect of short-time micronization by ball mill on structural and thermal properties of chemically modified sugar beet fibre, commercial sugar beet fibre (Fibrex^®^ 595), inulin and sucrose in order to compare micronization impact among structurally different dietary fibre. Apart from particle size reduction, the aim was to reveal structural and thermal changes in the corresponding fibre induced by short-time ball milling, which can further predict and tailor the use of dietary fibre in food products.

## MATERIALS AND METHODS

### Materials

Sugar beet pulp from a local sugar factory (Crvenka, Crvenka, Serbia) was treated with alkaline hydrogen peroxide according to the procedure described by Šoronja-Simović *et al.* (*8*) and subsequently dried in two stages (at 65 °C for 90 min and at 40 °C for 120 min) in a convective oven (Iskraterm 2 K; Iskra, Horjul, Slovenia), grinded (Thermomix®; Vorwerk, Wuppertal, Germany) and sieved (type SZ–1; ZBPP, Bydgoszoz, Poland) to obtain modified sugar beet fibre. Its fraction with particle size >315 µm was further micronized. Samples of commercial dietary fibre, inulin (Orafti® GR; BENEO-Orati S.A., Tienen, Belgium), sugar beet fibre (Fibrex® 595, particle size <0.125 mm; Nordic Sugar AB, Malmö, Sweden) and sucrose, were also examined.

### Ball milling treatment

Dietary fibre samples and sucrose were micronized in a planetary ball mill PM 100 (Retsch GmbH, Haan, Germany) equipped with ten stainless steel balls with 10 mm diameter placed in 50 mL cylindrical jar containing 5 g of the corresponding sample. Milling speed was set to 400 rpm with varying milling times (30 and 60 min). Accordingly, samples were marked with 0, 30 and 60 for the initial sample and samples after 30 and 60 min of micronization, respectively, and by the following designation: modified sugar beet fibre (MSBF), Fibrex (FI), inulin (IN) and sucrose (SU).

### Particle size distribution

The particle size distribution of the samples was determined by laser diffraction using Mastersizer 2000^®^ (Malvern Instruments Ltd., Malvern, UK) equipped with a Scirocco 2000 dry powder dispersion unit and particle refractive index of 1.52. Description of the particle size distribution was established by particle diameters corresponding to 10 (*D*_10_), 50 (*D*_50_) and 90% (*D*_90_) share of smaller to larger particles in cumulative particle volume. The width of the obtained distributions was depicted by span.

### Scanning electron microscopy analysis

Dietary fibre and sucrose morphology was observed by scanning electron microscopy (SEM) using Hitachi S-4700 (Hitachi Scientific Ltd., Tokyo, Japan). The sample preparation consisted of pre-coating with gold by the sputtering method. Applied magnification was ×70 for starting samples and ×500 for micronized samples.

### Fourier transform infrared analysis

The Fourier transform infrared (FTIR) spectra were recorded using a Thermo Nicolet AVATAR FTIR instrument (Thermo Fisher Scientific, Waltham, MA, USA). Pellets were prepared by co-grinding 10 mg of sample with 150 mg of potassium bromide and compressed with 10 tonnes using a hydraulic press. The FTIR spectra were recorded in the range of 4000‒400 cm^-1^ with a resolution of 4 cm^-1^ for 128 scans.

### X-ray diffraction analysis

X-ray diffraction analysis of the samples was conducted by X-ray diffractometer Bruker D8 Advance (Bruker AXS GmbH, Karlsruhe, Germany) at 40 kV and 40 mA with Cu Kα radiation (*λ*=1.5406 Å). Diffractograms were recorded in a 2*θ* scan range of 3–40° with a scan speed of 0.1°/min and step width of 0.01°.

### Thermal analysis

Differential scanning calorimetry (DSC) measurements were conducted using DSC 3+ (Mettler Toledo GmbH, Schwerzenbach, Switzerland) with integrated STARe software. An accurately weighed sample (10‒15 mg) was placed in an aluminium crucible and sealed with a lid. Measurements were performed in the synthetic air (velocity 100 cm^3^/min) within the temperature range 25‒300 °C and heating rate of 10 °C/min.

### Statistical analysis

The particle size distribution results in three replicates were subjected to one-way analysis of variance (ANOVA) using Statistica v. 14.0.0.15 software (*22*). Duncan’s multiple range test was applied for the determination of significant differences set at p≤0.05 between the mean values and homogeneous groups.

## RESULTS AND DISCUSSION

### Determined particle size distribution

**Fig. 1:**
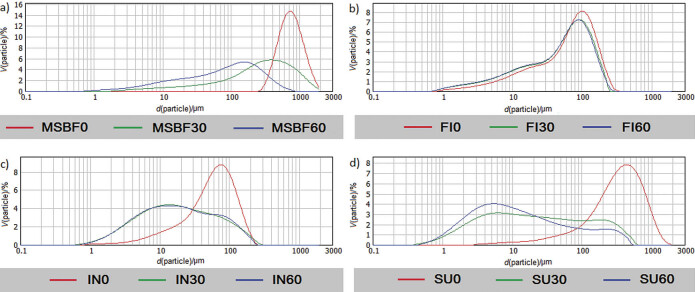
Particle size distribution of dietary fibre and sucrose before and after short-time micronization. MSBF=modified sugar beet fibre, FI=Fibrex 595, IN=inulin and SU=sucrose; *t*(milling)=0, 30 and 60 min

Characteristic parameters describing the particle size distribution of dietary fibre and sucrose after 30 and 60 min of micronization are presented in Table 1, while the particle size distribution of all samples is given in Fig. 1.

**Table 1:** Characteristic parameters describing the particle size distribution of dietary fibre samples and sucrose affected by short-time micronization

Sample	*t*(milling)/min	*D*_10_/µm	*D*_50_/µm	*D*_90_/µm		Span
MSBF	0	(440.4±3.7)^f^	(725.41±7.53)^f^	(1214±15)^g^	(1.06±0.01)^d^	
	30	(30.0±1.6)^d^	(286.1±3.2)^d^	(911.8±2.7)^f^	(3.08±0.02)^e^	
	60	(8.7±0.2)^b^	(84.4±1.3)^c^	(296.8±2.5)^d^	(3.42±0.08)^f^	
Fibrex 595	0	(10.0±0.1)^b^	(70.7±0.4)^bc^	(162.1±0.8)^a^	(2.15±0.01)^a^	
	30	(6.5±0.3)^ab^	(56.7±1.8)^b^	(143.6±2.1)^a^	(2.42±0.01)^b^	
	60	(6.2±0.1)^ab^	(54.8±0.1)^b^	(140.0±0.8)^a^	(2.44±0.04)^b^	
Inulin	0	(14.4±0.4)^c^	(61.4±1.2)^b^	(133.7±5.7)^a^	(2.03±0.07)^a^	
	30	(3.5±0.5)^a^	(17.4±0.7)^a^	(96.4±1.6)^b^	(5.33±0.02)^c^	
	60	(3.5±0.4)^a^	(17.4±0.4)^a^	(95.5±0.6)^b^	(5.28±0.02)^c^	
Sucrose	0	(70.1±5.5)^e^	(319.6±24.7)^e^	(748.9±56.6)^e^	(2.12±0.05)^a^	
	30	(2.3±0.2)^a^	(19.3±1.6)^a^	(222.1±1.7)^c^	(11.4±0.1)^g^	
	60	(2.0±0.3)^a^	(10.5±1.2)^a^	(152.8±1.2)^a^	(14.35±0.08)^h^	

Values represent the mean of three replicates. Mean values in the columns followed by different letters in superscript are significantly different (p<0.05) according to the Duncan’s multiple range test. MSBF=modified sugar beet fibre, *D*_10_, *D*_50_ and *D*_90_=particle diameters corresponding to 10, 50 and 90% share of smaller to larger particles in cumulative particle volume, Span=width of the particle size distribution

Samples of the same origin, modified sugar beet fibre and Fibrex 595, had different particle sizes, suggesting the effect of micronization and chemical modification. Greater particle size reduction of initial modified sugar beet fibre particles than of Fibrex 595 was observed due to a stronger attrition during milling. Modified sugar beet fibre was successfully reduced to a micron scale after 60 min of milling (*D*_50_=84.4 µm, reduction in median diameter 8.5 times, Table 1). Conversely, with the increase in milling time of Fibrex 595 from 30 to 60 min, a significant reduction in average particle size was not detected, while the overall particle size was reduced about 1.2 times (*D*_50_=54.8 µm, Table 1). A possible explanation could be that modified sugar beet fibre was more susceptible to the effect of attrition due to changes in the structure and weakened mechanical strength after lignin dissolution as a consequence of alkaline hydrogen peroxide treatment (*8*). A larger reduction in average particle size after short-time ball milling of grape pomace and fibre concentrate was previously reported by Bender *et al.* (*14*). Furthermore, Huang *et al.* (*6*) reported an average sugar beet pulp particle size at an ultra-micro scale (24.9 µm) after 5 h of superfine grinding. Nevertheless, a narrower and uniform particle size distribution of Fibrex 595 than of modified sugar beet fibre was indicated by the obtained span values. Similar span values obtained for Fibrex 595 suggested an even gradual attrition effect across all particles (Table 1 and Figs. 1a and 1b). Lower particle size and span values are favourable for specific applications (*12*) of dietary fibre such as bioactive compound excipients or emulsion stabilisers (*21*), aiming to enhance the possibility of homogenization with other ingredients and dispersibility within the food matrix (*11*).

Reduction in the size of inulin particles with prolonged milling time was neglectable since *D*_50_ values obtained after 30 min were almost even to those obtained after 60 min of micronization (Table 1). However, smaller particle sizes were noted than of both samples originating from sugar beet pulp, indicating a greater milling effect probably due to different chemical compositions and predominantly amorphous structure of commercial inulin types (*23*). Increased span values for inulin suggest the presence of a higher amount of fine particles but also an uneven micronization effect, which results in broadening of the particle size distribution curve (Fig. 1c).

The effect of ball milling on particle size reduction was strongly pronounced on sucrose where the median diameter decreased from 319.6 to 19.3 µm after only 30 min of milling (*D*_50_ reduction 16.5 times). Increasing milling time caused further reduction in particle size (*D*_50_ reduction 33 times, Table 1). However, the corresponding decrease in particle size was not evenly distributed. Smaller sucrose particles were more susceptible to the attrition and hence were the first to be further micronized. This increased the number of very fine sucrose particles (~2 µm), which is reflected in a very wide particle size distribution as indicated by high span values (Fig. 1d and Table 1). Additionally, the observed rise in sucrose span values with increasing milling time was the most pronounced compared to other samples. It is supposed that the span value would continue to rise with further milling due to high local mechanical energy impute leading to temperature elevation and consequently melting of the outer molecule layers (*24*), hence increasing stickiness. The corresponding observation demonstrates that ball milling is not an appropriate method for uniform reduction in particle size of sucrose crystals and is more applicable for lignocellulosic material such as sugar beet pulp. Furthermore, short-time milling applied herein proved to be effective for particle size reduction of modified sugar beet fibre and Fibrex 595 to micron-scale, which is favourable for decreasing processing costs.

### Determined morphology by scanning electron microscopy

**Fig. 2:**
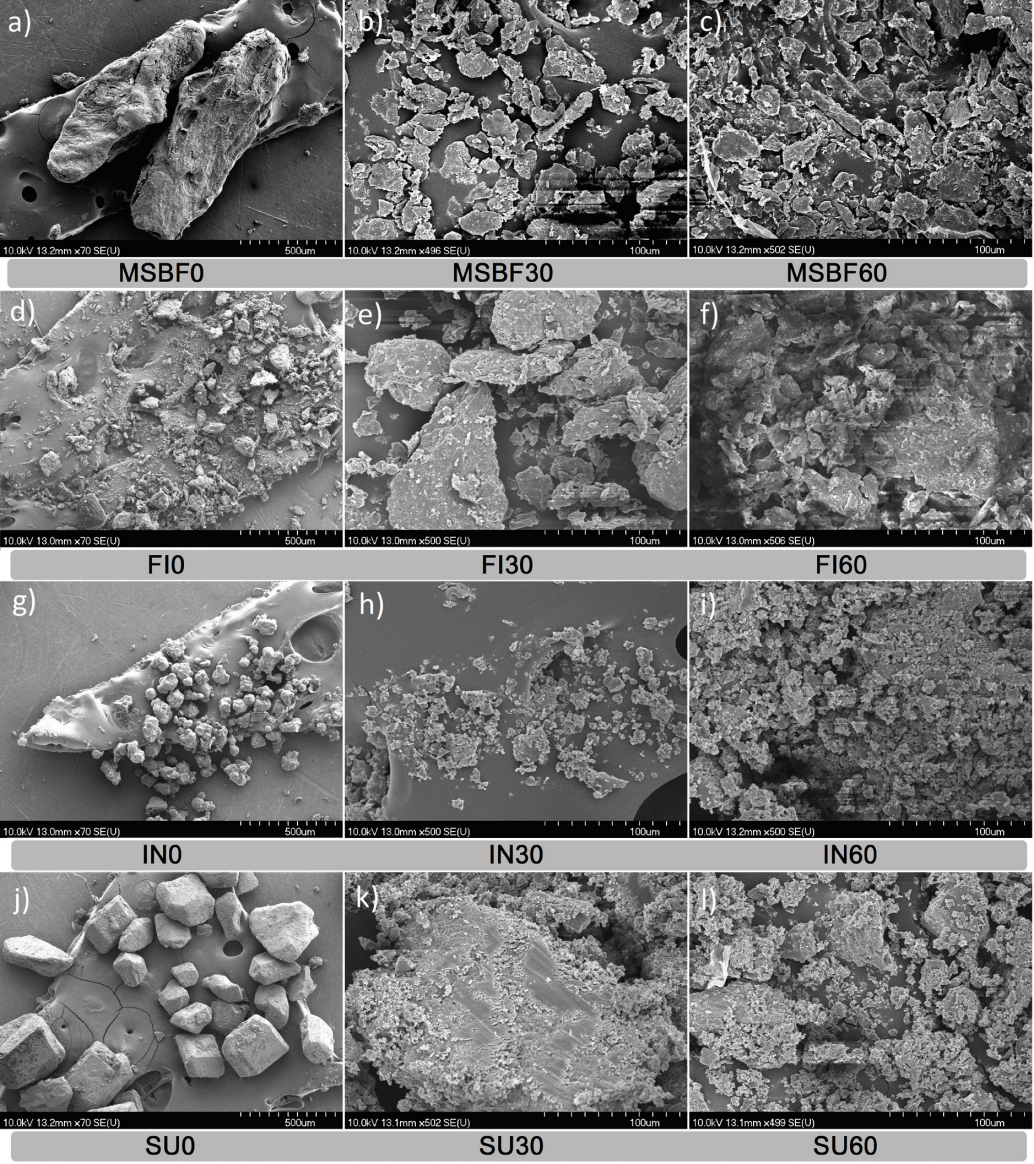
Scanning electron micrographs of the dietary fibre samples and sucrose at ×70 and ×500 magnification showing the effect of short-time micronization. MSBF=modified sugar beet fibre, FI=Fibrex 595, IN=inulin and SU=sucrose; *t*(milling)=0, 30 and 60 min

Reduction in particle size of the samples is also reflected in the morphology and matrix disruption as evidenced by the obtained scanning electron microscopy (SEM) micrographs (Fig. 2). The already altered lignocellulose structure of sugar beet pulp after alkaline hydrogen peroxide modification (conductive tissue segment disruption related to lignin, cellulose and hemicellulose fragmentation) (*6*) was further modified/changed by the attrition during ball milling. Rounded edge particles with irregular shape and size were observed after the micronization of modified sugar beet fibre with an increasing number of small fragments as the milling time prolonged (Figs. 2a-2c) suggesting further fracture of the rigid structure. The corresponding fragments are usually related to lignin and cellulose moieties formed as a consequence of intermolecular bond breakage caused by milling (*14*). Noticeable particle size reduction was visible on modified sugar beet fibre and sucrose micrographs as demonstrated by the particle size distribution results. These observations were confirmed by the increase in the span values of the corresponding fibre (Table 1). The surface of the modified sugar beet fibre particles was slightly coarse with a number of rifts and without visible pores. Sharper edges were observed on micronized Fibrex 595 particles than on modified sugar beet fibre with similar furrowed surfaces interspersed with small fragments of diverse shapes (Figs. 2d-2f).

Lumps with round edges of inulin particles turned to irregularly shaped shards with sharp edges prone to aggregation, as observed in micrographs, especially after 60 min of milling (Figs. 2g-2i). A longer milling time increases the temperature of the sample as well as its amorphous portion. If the temperature of glass transition is exceeded, agglomeration of inulin particles could occur due to increased stickiness (*25*).

Distinctive cubic crystal structure of sucrose with clear surface and perfectly defined edges was observed in the micrographs before milling (SU0, Fig. 2j). Loss of properly defined edges, the step-like structured surface (*24*) of remaining parts of crystals with attached and free irregularly shaped fragments in varying size were detected after micronization (SU30 and SU60, Figs. 2k and 2l). The presence of a large number of different fragments was also confirmed by higher span values than in dietary fibre samples, implying a very wide particle size distribution (Table 1 and Fig. 1d). This could be attributed to faster crystal destruction due to lower sucrose rigidity than dietary fibre and hence enhanced manifestation of attrition during milling.

### Functional groups and bonds determined by Fourier transform infrared analysis

**Fig. 3:**
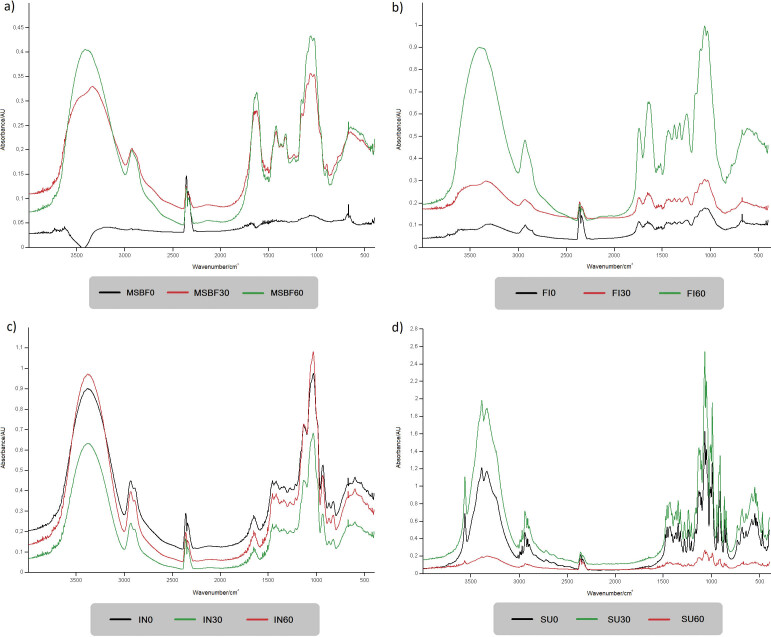
FTIR spectra of dietary fibre and sucrose before and after the short-time micronization. MSBF=modified sugar beet fibre, FI=Fibrex 595, IN=inulin and SU=sucrose; *t*(milling)=0, 30 and 60 min

The basic structure of dietary fibre and sucrose in the solid state was assessed through Fourier transform infrared (FTIR) analysis. Differences among the studied carbohydrates as well as the influence of micronization were determined by the identification of the obtained band patterns presented in Fig. 3. Band assignments to the corresponding functional group or bond are summarized in Table 2 (*26*-*38*). Three main regions were observed on the spectra regardless of the sample, O–H stretching, C‒H stretching and the fingerprint region. The most informative and hence reliable for distinction between carbohydrates, including ones comprising glucosyl units, is the fingerprint region (*26*, *36*). According to the spectra, the general spectral profile remained unchanged in all samples regardless of the applied micronization time, suggesting that the main sample structure was retained. Nevertheless, a decrease in particle size led to variations in the band intensity (absorbance) (Figs. 3a-3d), and sporadic shifts in band positions (wavenumber) (FI, Fig. 3b). With an increase in milling time, band intensity increased for dietary fibre (Fig. 3a-3c), while the opposite effect was observed for sucrose (Fig. 3d). Broad bands in the range 3600‒3000 cm^-1^ centred at approx. 3370‒3330 cm^-1^ depending on the sample, corresponds to the O‒H stretching vibrations of present OH groups within glucosyl units of sucrose and polysaccharides (*26*, *28*). A shift of the centred band in the corresponding region towards higher wavenumbers was noted for Fibrex 595 after 60 min of micronization (FI60, Fig. 3b). Sharp isolated band at ~3555 cm^-1^ was noted in sucrose spectra which corresponds to the O‒H stretching in fructosyl unit (*26*, *27*) and its intensity increased with micronization time. Furthermore, prolonged micronization induced the rise in intensity of two bands in the 3600‒3000 region of sucrose. Bands observed in the corresponding region reflect vibrations of OH groups due to variations in intra- and intermolecular hydrogen bonds, distances between two oxygen atoms and angles between the OH group and oxygen atom (*39*). The observed wavenumber shifts were attributed to the weakening or disruption of hydrogen bonds induced by the applied mechanical force during micronization accompanied by a rise in sample temperature (*14*-*16*, *40*).

**Table 2:** Vibrational band assignments corresponding to the FTIR spectra of dietary fibre and sucrose affected by short-time micronization.

MSBF	Fibrex 595	Inulin	Sucrose	Bond vibration	Reference
			3555-3550	O‒H stretching, fructosyl unit	(*26*, *27*)
3600-3000, 3395	3650-3000, 3370, 3330	3600-3000, 3365	3500-3000, 3370	O‒H stretching, glucosyl unit	(*28*) (*26*)
			3325-3323	O‒H stretching, fructosyl unit	(*26*)
			2980-2975	C–H stretching, glucosyl unit	(*26*)
			2940	C–H symmetric stretching, methylene	(*26*)
		2935-2930		C‒H asymmetric stretching, methylene	(*29*, *30*)
2930-2925				C‒H stretching, hydrocarbon chain	(*31*, *32*)
			2920	C‒H symmetric stretching, methylene	(*27*)
	2910-2900			C‒H stretching, hydrocarbon chain	(*33*)
		2880		C‒H symmetric stretching, methyl	(*30*)
	1740-1735			C = O stretching, ester bond, aldehydes, ketones	(*34*, *35*)
		1640		C = O stretching, amide I, protein H-O-H stretching	(*36*, *37*)
	1630-1635			C = O stretching, amide I, pectin, H-O-H stretching	(*28*) (*35*)
1625				C = O stretching, amide I, pectin	(*28*)
	1510			C = C stretching, aromatic ring, lignin	(*31*, *35*)
			1465	C‒H scissoring, methylene	(*27*)
		1455-1450		C‒H out-of-plane bending, methyl, methylene	(*36*)
	1435-1430		1430	C‒H in-plane bending, methylene C‒H rocking	(*26*, *27*, *38*)
1415-1410		1410-1408		C‒H in-plane bending, methylene	(*30*, *35*)
	1375			C‒H bending, hydrocarbon chains	(*31*, *35*)
	1345-1340		1345-1340	C‒H rocking, methylene	(*27*)
		1334		C‒H in-plane bending, methylene	(*30*)
1323-1320				C‒H bending, cellulose	(*31*)
			1270-1275	C‒H rocking, methylene	(*27*)
	1249-1247			C‒O stretching, lignin	(*31*)
					
			1230	C‒H bending, methylene O‒H in-plane bending	(*27*) (*26*)
	1150			C-O-C stretching	(*31*)
1123-1120		1120-1115	1125-1120	C‒O and C-O-C stretching	(*30*)
	1070			C‒O stretching	(*31*)
1050-1055	1053-1051		1053-1050	C‒O stretching	(*27*, *37*)
1020	1029	1030-1020		C-O-H stretching, C-O-C stretching out-of-plane cyclic ring	(*36*)
			988	C‒O stretching, glucosyl unit	(*26**,**27*)
		940-935		C-O-C stretching cyclic ring, exocyclic glicosidic bond	(*36*)
			910	C‒H twisting, methylene	(*26*, *27*)
900				C-O-C stretching cyclic ring, exocyclic glycosidic bond C-O-H stretching	(*36*)
		879		C-O-C stretching cyclic ring, exocyclic glycosidic bond C-O-H stretching	(*36*)
			858-855	C‒H twisting, methylene	(*27*)
			730-725	C‒O stretching, in-plane ring deformation, glucosyl unit	(*26*, *27*)
			630	In-plane ring deformation	(*27*)
			580	C-O-C in-plane bending, fructosyl unit	(*26*)
			540-535	Ring deformation, glucofuran	(*26*, *27*)
			475-470	C-O-C in-plane bending, fructosyl unit	(*26*)

MSBF=modified sugar beet fibre

Bands in the range 3000‒2800 cm^-1^ designated the C‒H stretching vibrations present in the examined dietary fibre and sucrose (Table 2). C‒H stretching of methyl and methylene groups within the cellulose, hemicellulose and pectin hydrocarbon chains as sugar beet pulp constituents were detected in modified sugar beet fibre and Fibrex 595 at 2930‒2925 cm^-1^ and 2910‒2900 cm^-1^, respectively (*31*-*33*). Asymmetric and symmetric C‒H stretching of methyl and methylene groups within inulin structure were noticed at 2935‒2930 and 2880 cm^-1^, respectively (*29*, *30*). Characteristic stretching of the C‒H bond in the sucrose glucosyl unit was detected at 2980‒2975 cm^-1^ (*26*) alongside the symmetric C‒H stretching of methylene groups at 2940 and 2920 cm^-1^ (*26*, *27*). Stronger intensity of the corresponding bands regardless of the sample was observed after micronization, suggesting an increased exposure and accessibility to the present functional groups of saccharides, as previously observed for olive pomace and soybean residue (*15*, *19*).

Sample diversity was further depicted within the fingerprint region. The main differences observed between the modified sugar beet fibre and Fibrex 595 were in the bands that indicated the presence of pectin and lignin (Table 2). The bands at ~1740, ~1510 and ~1249 cm^-1^ corresponding to C=O stretching, C=C stretching and C‒O stretching, respectively, within the lignin structure were noticed only in Fibrex 595 and their intensity increased after micronization. The C=O stretching at ~1740 cm^-1^ is also an indicator of the presence of the esterified carboxyl groups in pectin, a sugar beet pulp constituent (*41*). The absence of the corresponding bands in the modified sugar beet fibre spectra could be associated with the fragmentation of lignin as well as with the potential disruption of ester bonds among lignin and polysaccharides as a consequence of the conducted alkaline hydrogen peroxide treatment (*8*, *42*).

Samples originating from sugar beet pulp (modified sugar beet fibre and Fibrex 595, Figs. 3a and 3b) also exhibited overlapping bands of amide I and water in the range 1700‒1600 cm^-1^, confirming the presence of a proteinaceous moiety in the pectin structure (*28*) which was not affected by micronization. Weaker bands detected in the range 1465‒1230 cm^-1^ regardless of the sample were associated with various C‒H bending vibrations predominantly in the methylene groups of monosaccharide units (sucrose) and hydrocarbon chains (dietary fibre) (Table 2). The strongest absorption bands for modified sugar beet fibre, Fibrex 595 and sucrose at ~1053 cm^-1^ were even more pronounced after micronization and ascribed to C‒O stretching in carbohydrates (*37*). Additionally, C‒O‒H and C‒O‒C stretching vibrations associated with wavenumber 1029 cm^-1^ were present in all dietary fibre samples and the strongest displayed band was detected in inulin (*36*). Stretching vibrations of C–O, C–O–C and C–O–H of the cyclic ring in all samples were observed in the range of 988‒879 cm^-1^ (Table 2). Below 858 cm^-1^, various stretching and bending vibrations of glucosyl and fructosyl unit bonds were detected in the sucrose spectrum (Table 2). Furthermore, a decrease in the band intensity of sucrose after 60 min of micronization was noticeable (SU60, Fig. 3d). Similarly, Zhao *et al.* (*43*) observed a common reduction of bands intensities for ginger powder with particle size decrease. As a consequence of the applied mechanical force during grinding, the intramolecular hydrogen bonds in the amorphous region of cellulose and hemicellulose break (*14*, *16*), inducing also increased exposure of functional groups (*15*). This reflects in the variations of the absorbance and wavenumber of the spectra. However, the impact of short-time micronization on the main functional groups in the samples was not detected since they remained unchanged.

### Structure determined by X-ray diffraction analysis

**Fig. 4:**
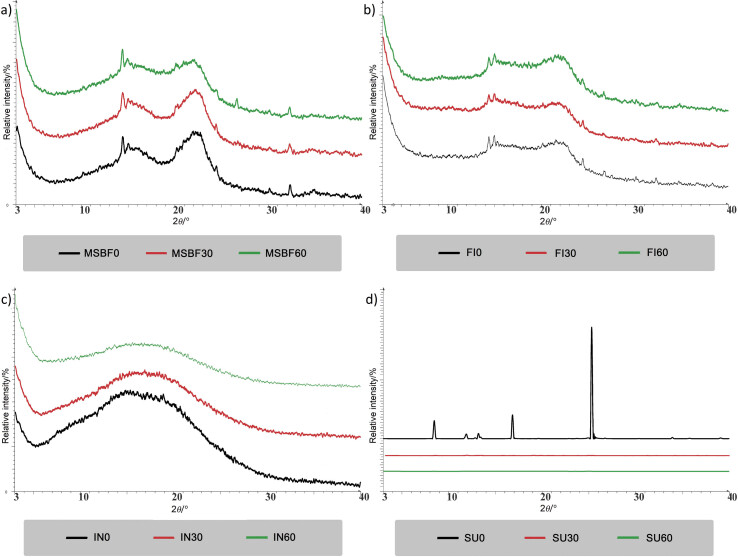
X-ray diffraction patterns of dietary fibre and sucrose subjected to short-time micronization. MSBF=modified sugar beet fibres, FI=Fibrex 595, IN=inulin and SU=sucrose; *t*(milling)=0, 30 and 60 min

X-ray diffractograms (XRD) of dietary fibre and sucrose subjected to different milling times are shown in Fig. 4.

Visually similar XRD patterns were obtained for modified sugar beet fibre and Fibrex 595, suggesting a semicrystalline structure with a prominent peak at around 2*θ*=22° and two lower peaks at approx. 2*θ*=14.5‒15° and 34° (Figs. 4a and 4b) as characteristic of cellulose I (*44*). Sharper diffraction peaks were noticed for modified sugar beet fibre than for Fibrex 595 as a consequence of the alkaline hydrogen peroxide treatment. Namely, partial removal of hemicellulose and lignin, and the hydrolysis of the cellulose amorphous regions suggests a higher degree of crystallinity (*35*). Nevertheless, the conducted micronization induced to a certain extent an increase in the modified sugar beet fibre and Fibrex 595 diffraction peak intensity and sharpness, but also peak widening (Figs. 4a and 4b). Accordingly, it is assumed that the applied mechanical force during micronization primarily affects the amorphous regions of the corresponding samples (*15*, *16*), as evidenced by FTIR analysis. However, potential changes in the crystalline structure of sugar beet pulp after 5 h of superfine grinding were also reported by Huang *et al.* (*6*). For inulin, a broad diffraction peak or broad halo pattern in the range 2*θ*=6‒25° was noticed (IN, Fig. 4c) and it is characteristic of an amorphous sample (*45*). With prolonged micronization time (60 min) the corresponding halo pattern became more flattened and peak width increased, suggesting the presence of a more diverse distance between the present atoms due to the applied mechanical force. Visually similar halo patterns of amorphous inulin with *w*(water)=0.9 and 15.7 g/100 g dry inulin were reported by Ronkart *et al.* (*45*). Conversely, the extremely sharp peaks appearing in sucrose diffractograms were definite conformation of a pure crystalline structure. The peaks with the highest relative intensities were detected at 2*θ*≈8.5°, 17° and 25.3° in the starting sample and further greatly diminished and/or disappeared with an increase in micronization time (SU, Fig. 4d). Accordingly, a transformation from crystalline to amorphous structure was observed in sucrose samples, implying the destruction of sucrose crystals (*46*), as visible in SEM micrographs (Figs. 2k and 2l).

### Thermal characteristics determined by differential scanning calorimetry

**Fig. 5:**
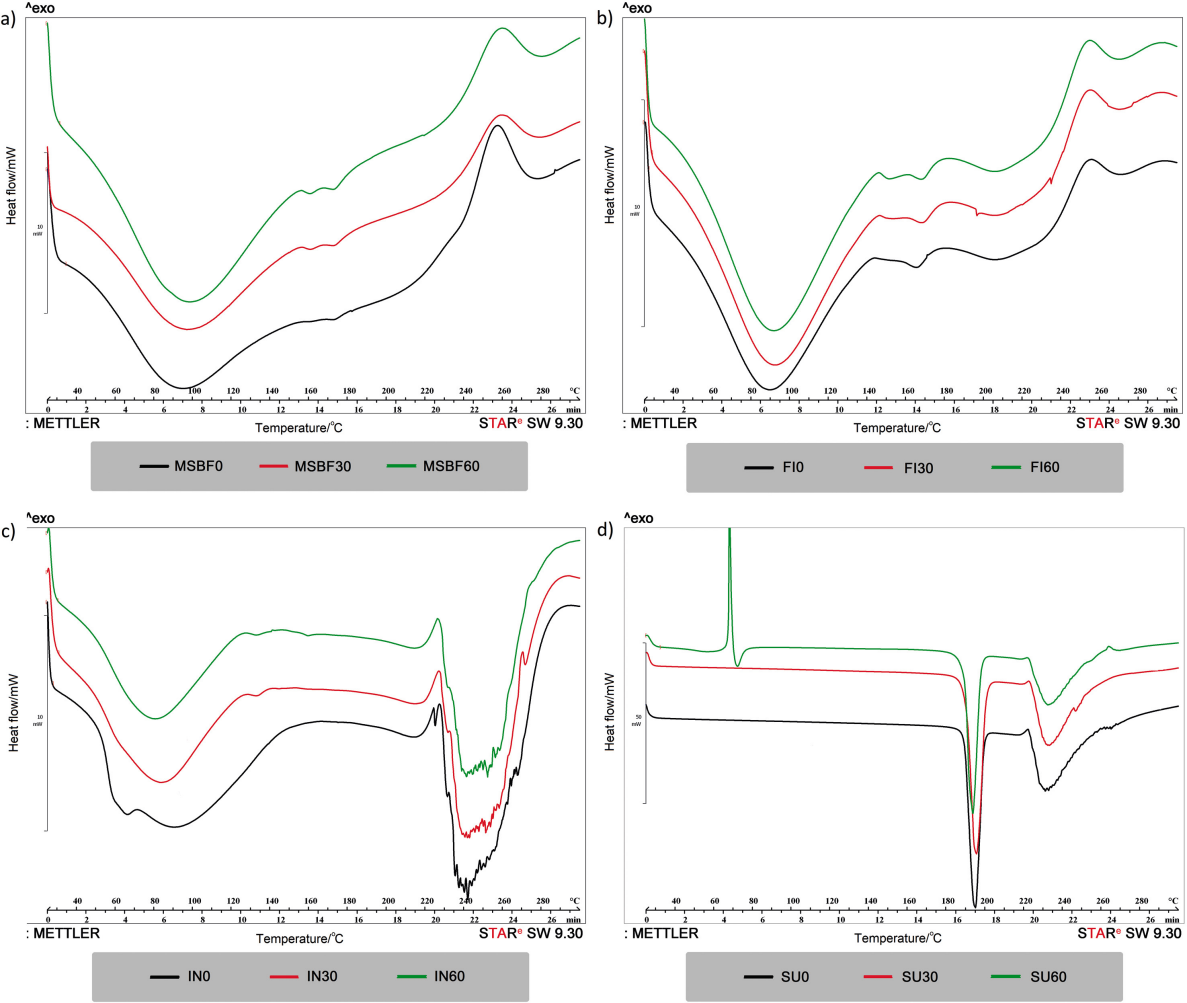
Differential scanning calorimetry (DSC) thermograms of short-time micronized dietary fibre and sucrose. MSBF=modified sugar beet fibre, FI=Fibrex 595, IN=inulin and SU=sucrose; *t*(milling)=0, 30 and 60 min

To assess the effect of short-time micronization on the thermal behaviour of dietary fibre and sucrose, differential scanning calorimetry (DSC) thermograms at a 10 °C/min heating rate are shown in Fig. 5, while the corresponding thermal parameters are summarized in Table 3. Regardless of the sample, the obtained thermograms suggest the occurrence of endothermic reaction. An increase in micronization time usually led to similar or higher peak temperatures (*t*_p_) in all samples except inulin, where the opposite tendency was observed (Table 3). Visually similar thermograms were obtained for modified sugar beet fibre and Fibrex 595 with major sections representing an endothermic peak in the temperature range 43.64‒149.82 °C (Figs. 5a and 5b), primarily attributed to the free water evaporation (*47*). Recorded *t*_p_ for modified sugar beet fibre and Fibrex 595 before micronization was 93.10 and 82.65 °C, respectively, and it increased with the applied micronization time as well as the specific enthalpy change (Table 3). Nevertheless, previously reported higher peak temperatures for sugar beet pulp (125‒142 °C) (*6*, *48*) suggest that the applied mechanical force herein was strong enough to release the present water without exposing the groups susceptible to change. Accordingly, modified sugar beet fibre and Fibrex 595 could be regarded as thermostable and hence applicable as excipients in emulsions or suspensions even at elevated temperatures (*49*).

**Table 3:** Thermal properties of dietary fibre and sucrose subjected to short-time ball milling

Sample	*t*(milling)/min	*t*_o_/°C	*t*_p_/°C	*t*_c_/°C	Δ*h*/(J/g)
MSBF	0	47.67	93.10	145.03	-143.03
	30	47.67	96.43	148.20	-195.10
	60	46.42	95.12	149.82	-215.80
Fibrex 595	0	43.64	82.65	129.45	-160.22
	30	47.22	91.04	139.12	-159.94
	60	47.65	90.36	138.47	-157.00
Inulin	0	77.41	88.65	145.21	-188.68
	30	43.46	82.72	121.39	-146.37
	60	44.35	79.41	122.17	-133.13
Sucrose	0	188.89	192.23	198.95	-132.21
	30	189.34	192.74	199.90	-139.38
	60 Peak 1 Peak 2 Peak 3	67.29 70.03 188.84	69.44 72.03 191.24	69.72 75.91 197.48	31.09 -12.55 -140.09

MSBF=modified sugar beet fibre, *t*_o_=onset temperature, *t*_p_=peak temperature, *t*_c_=end set (conclusion temperature), Δ*h*=specific enthalpy change of transition

All inulin samples, regardless of micronization time, exhibited a broad endothermic peak (Fig. 5c). For IN30 and IN60, the endothermic peak was obtained in the temperature range from 43 to 122 °C (Table 3). Slight variation in the endothermic peak appearance was observed for the starting inulin sample (IN0) in terms of increased broadness and dual peak presence in the temperature range of 77.41-145.21 °C (Table 3). The corresponding peak was assigned to water evaporation from the samples and was also previously detected by Panchev *et al.* (*50*) and Ronkart *et al.* (*51*). Furthermore, regardless of the sample, thermal degradation was observed in the temperature range 220‒270 °C, as previously reported (*51*).

A large endothermic peak was detected in sucrose thermograms, regardless of micronization time at onset temperature (*t*_o_) of nearly 189 °C (Table 3), followed by a smaller endothermic peak at approx. 230 °C, which represents characteristic of crystalline sucrose. The first peaks were associated with sucrose crystal lattice melting, namely loss of crystalline structure due to the applied heat (*52*, *53*). The second ones were attributed to sucrose decomposition due to cleavage of disaccharide bonds, followed by water elimination from monosaccharides and transformation towards volatile and nonvolatile aroma compounds (*46*, *54*).

Nevertheless, alteration of the DSC curve for SU60 sample was reflected through the presence of two more peaks at lower temperatures (Table 3 and Fig. 5d), suggesting the existence of an amorphous structure obtained after prolonged micronization (*24*), which is in accordance with the particle size (Table 1) and XRD results (SU30 and SU60, Fig. 4d). The exothermic peak could be associated with crystallization, while the origin of the endothermic peak could be ascribed to accelerated release of water entrapped within the mother liquor occlusions in sucrose crystals induced by micronization (*52*, *53*).

## CONCLUSIONS

Short-time micronization by planetary ball mill was employed for particle size reduction of modified and commercial sugar beet fibre, inulin and sucrose alongside monitoring the corresponding impact on structural, thermal and physical changes. Particle size reduction by micronization was the most effective for modified sugar beet fibre and sucrose, where the reported decrease in median diameter was approx. 8.5 and 33 times, respectively. Nevertheless, the conducted micronization reflected unfavourably on inulin and sucrose by inducing yield losses and high span values. Regardless of the sample, increased exposure and accessibility of the present functional groups were noticed as a consequence of the applied mechanical force, implying the intramolecular hydrogen bond breakage. Additionally, the mentioned mechanical force induced changes primarily in the amorphous regions of corresponding samples, while the thermostability of modified and commercial sugar beet fibre remained unaffected by the applied force. Short-time micronization by ball mill was recognised as an effective way for improving the bioavailability of sugar beet fibre as well as enabling their application as excipients in food products. Additionally, high suitability for industrial scale-up of the process is enabled due to the cost-effectiveness and eco-friendly approach.
